# Tourism Competitiveness Evaluation: Evidence From Mountain Tourism in China

**DOI:** 10.3389/fpsyg.2022.809314

**Published:** 2022-03-31

**Authors:** Qian Cao, Md Nazirul Islam Sarker, Dian Zhang, Jiangyan Sun, Teng Xiong, Jieying Ding

**Affiliations:** ^1^Research Center of Eco-cultural Tourism in Western Hubei, Hubei Minzu University, Enshi, China; ^2^School of Political Science and Public Administration, Neijiang Normal University, Neijiang, China; ^3^Hubei Enshi College, Enshi, China; ^4^Penshui County Development and Reform Commission, Chongqing, China; ^5^Institute of Ethnology and Sociology, Southwest Minzu University, Chengdu, China

**Keywords:** tourism psychology, Enshi Autonomous Prefecture (EAP), tourism industry, tourism competitiveness, competitiveness evaluation

## Abstract

The evaluation of tourism competitiveness is an important tool for analyzing the potential of tourism in a specific context. Enshi Autonomous Prefecture (EAP) in China is selected as a case through which to explore the potential of mountain tourism and its competitiveness in the tourism industry. This study develops EAP’s mountain tourism competitiveness model focusing on three criteria: core competitiveness of mountain tourism, the economic environment’s competitiveness, and infrastructure competitiveness. Context-specific customized evaluation index has been applied to data collected from EAP Statistical Yearbook for 2005–2014. The study reveals that the value of EAP’s mountain tourism core competitiveness, economic and environmental competitiveness, and infrastructure competitiveness are 84.292, 13.4, and 2.308%, respectively. When tourism core competitiveness is increased by one unit, EAP’s mountain tourism competitiveness will increase by 0.84292 units. Similarly, when economic environment competitiveness is increased by one unit, EAP’s mountain tourism competitiveness will increase by 0.134 units. EAP’s mountain tourism competitiveness increases by 0.02308 units when infrastructure competitiveness increases by one unit. The major reasons for low levels of competitiveness were lack of awareness of the county authority, a low level of cooperation, and weak infrastructure. The recommendations from the study’s findings are as follows. Firstly, the county authority should appropriately improve the relationship between competition and cooperation, maintaining cooperation in competition, and competition in cooperation. Secondly, the county authority should strengthen communication by establishing an effective coordinated mechanism. Thirdly, the county authority should improve the sense of cooperation and jointly develop the mountain tourism market. Fourthly, the county authority should improve the construction of tourism infrastructure and break down the barriers to tourism cooperation. The study’s findings help develop a “win-win” cooperation mechanism within the competition and support the sustainable development of the mountain tourism industry while reducing poverty and promoting the revitalization of the mountains of China.

## Introduction

Mountain tourism is considered to be a key tool for poverty reduction, economic development, and environmental management. Rapidly increasing the level of the mountain tourism industry can enhance the progress of society, national income, the demand for tourism, and overall economic development (Wang et al., 2013). The tourism industry also plays an important role in promoting cultural exchange, employment, and regional economic development (Liu et al., 2022). At present, the tourism industry has become the fastest growing industry and is one of the important pillars of world economic activities, with this highly recognized in all countries worldwide (Shen et al., 2019). China also places great importance on developing the mountain tourism industry, strengthening various measures to promote the development of this industry and encouraging mountain tourism competition between regions (Zeng et al., 2022). Tourist destination competition usually affects the redistribution of market opportunities (Patton, 1985; Shi et al., 2016). The associated challenges include scientifically evaluating the competitiveness of mountain tourism destinations and the formation of measures to enhance tourism competitiveness (Nazmfar et al., 2019). In contrast to other tourism sectors (such as spa, heritage, and sightseeing tourism), Mountain tourism allows visitors to pursue wellness and associated passions (Zeng et al., 2022). Because of its recreational, detaching, healing, and sporting characteristics, the mountain attracts the largest flows of tourists, and it is becoming a key social and educational component for the community (Bacoş and Gabor, 2021). Considering the importance, this study focuses on the potential of mountain tourism and its competitiveness in the tourism industry.

Tourism climate focuses on the physical, thermal, and aesthetic variables. Most physical and aesthetic qualities are subjective, which plays a significant role in tourism. China’s tourist business has grown into an all-encompassing sector encompassing a wide range of activities. Tourism has emerged as a new engine for developing the national economy in China. In contrast to the falling trend in the number of incoming tourists to China, the quality of Chinese tourism services has remained “generally constant with steady growth” in recent years (Peng and Yuan, 2019). As a result, the slow growth of inbound tourism in recent years has had only a negligible impact on the quality of tourism services. Intuitively, the confluence of increased haze and decreased incoming visitor numbers may explain the slow growth of inbound tourism, and the link between the two has piqued the interest of academics. Haze has a detrimental effect on inbound tourism traffic in China. Furthermore, haze has impacted inbound tourism and domestic tourism, which has seen a drop in demand and market growth, exacerbating the tourist sector’s cyclical oscillations. The Chinese government recently suggested the development strategy of constructing an ecological civilization and the grand objective of building a “beautiful China” to alleviate the threat of significant environmental degradation to the Chinese economy’s long-term viability (Zeng et al., 2022). Therefore, an investigation on tourism competitiveness is urgent to develop the industry.

Several studies have been conducted on the evaluation index system of tourism competitiveness. For example, Li and Du (2021) investigated the coupling coordination relationship between culture and tourist flow in China and used the competitiveness evaluation index for measuring tourism competitiveness. Gao et al. (2021) conducted a study on tourism competitiveness and sustainability in China by using an evaluation index approach. Haahti (1986) formulated this evaluation index system, evaluated Finland’s tourism competitiveness, and proposed a series of measures to consolidate Finland’s tourism competitiveness. Kozak and Rimmington (1999) divided the indicators of tourism competitiveness into hard indicators and soft indicators, thus being able to measure the tourism competitiveness of tourism destinations by combining hard indicators with soft indicators. Sánchez et al. (2006) used social information technology (IT), national policy, and tourism talent in their evaluation index system of tourism competitiveness. The brand image of tourism is an extremely important factor in evaluating tourism competition (Law, 2001; Law, 2007; Greene et al., 2007; Chon et al., 2010; Zhang et al., 2011; Zhang and Lu, 2012). Zhang and Lu (2012) selected evaluation indexes from the following four aspects: factor competitiveness, market competitiveness, management competitiveness, and development competitiveness. Su et al. (2003) established an index system from the perspectives of urban tourism competitive performance, competitive potential of urban tourism, and urban tourism competitive environment support. Zhang and Zhou (2005) selected evaluation indexes using four aspects: tourism development scale, outbound tourism ability, tourism organization ability, and tourism reception ability, using a province as their study area.

Tourism competitiveness is a key issue for governments and destinations seeking a competitive edge in the ever-changing global tourist industry. The relative competitiveness of tourism sites influences their performance in global marketplaces (Sedlacek et al., 2022). Attracting tourists to locations has gotten more difficult as global tourism market growth has slowed and market shares have shifted. As a result, the tourist competitiveness of locations has received more attention. The strength or capacity of a place to give a great experience to tourists is at the heart of tourism competition (Zeng et al., 2022). The issue of tourism competitiveness is critical for countries that want to monitor and perform effectively in the global tourism sector. Understanding a country’s tourism competitiveness is critical for policymakers and a significant task for professionals in producing evidence to support decision-making.

Tourism competitiveness has become a hotspot of theoretical and practical research. Scholars have conducted a few studies on the basic connotation (Zhang et al., 2011; Deng et al., 2019), management (Maso et al., 2016), influencing factors (Wang et al., 2013), rurality (Cvelbar et al., 2016; Shen et al., 2019), and evaluation methods (Mendola and Volo, 2017; Jiao et al., 2018). These scholars have achieved rich results, but some issues still need to be solved urgently. Firstly, evaluations have been done regarding the competitiveness of different regional tourism destinations (Lopes et al., 2018; Sedlacek et al., 2022). Existing studies mostly focus on national, provincial, and developed urban tourism destinations (Lee et al., 2010; Chen et al., 2011), thus belonging to macro-evaluation research. However, few studies have been conducted on the micro-evaluation of tourism destinations for underdeveloped regions, especially for regions in which ethnic minorities live, as in the Western mountains of China. Secondly, the existing evaluation index system is relatively macroscopic, incomplete, and not specific; thus, it is not suitable for the systematic evaluation of the competitiveness of local or regional tourism destinations. Thirdly, the existing research is mainly from the perspective of competitiveness but lacks the perspective of cooperation in exploring the competitiveness of tourism destinations. This study intends to fill the research gap by addressing a couple of questions, like what is the potential of mountain tourism? Is mountain tourism being competitive in the tourism industry in China? And what will be the context-specific measurement of mountain tourism competitiveness? These issues are becoming increasingly important to the government, academics, and the general public. It is critical to construct a technology-based system for evaluating mountain tourism competitiveness, to statistically evaluate and scientifically identify obstacles impeding actual demands, as well as the simultaneous pressures of the authority and competition (Gao et al., 2021). Tourist destinations need both competition and cooperation (Zhang et al., 2022). To fundamentally enhance the competitiveness of tourist destinations, it is necessary to strengthen cooperation between these destinations, such as in the mountain tourism industry in China, and achieve “win-win” cooperation.

Enshi Autonomous Prefecture (EAP), an important tourist destination in Western China, has eight counties and is located in China’s Western mountains. As tourism resources are mainly concentrated in mountain areas, mountain tourism is the main form of tourism. The EAP government has given higher priority to developing the mountain tourism industry to make it the prefecture’s leading industry. An analysis and evaluation of EAP’s mountain tourism competitiveness can help clarify the attractiveness of each region’s mountain tourism industry and identify the direction for its development. Therefore, the development of evaluation indicators and models, the analysis of key competitiveness factors, and the development of future directions are conducive to maintaining long-term competitive advantage. These drivers can promote the sustainable and healthy development of EAP’s mountain tourism industry. Therefore, the aim of this study is to construct an evaluation model of tourism competitiveness for the mountain tourism industry. The rest of the manuscript is arranged as follows. Section “Literature Review” presents the literature review, sections “Materials and Methods” and “Results” respectively describe the methodology, results, and discussion, while section “Discussions” concludes the manuscript with recommendations.

## Literature Review

Rapid urbanization in China is stimulating its citizens to develop feelings for nature and to spend the maximum amount of their leisure time in tourist destinations. Mountain tourism provides not only a place of entertainment but also acts as a key tool for developing the mainstream economy. Tourism competitiveness is defined as the ability of a tourist destination to attract and satisfy potential tourists (Enright and Newton, 2004; Zhang et al., 2011). Ritchie and Crouch (2000) explained the concept of tourism competitiveness in terms of the value addition from tourism destination development in a country. Pearce (1997) believed that the competitiveness of tourist destinations depends on the method of evaluation of their development. Tourism competitiveness is also the ability of tourism competitors to obtain comprehensive benefits from international and domestic perspectives. Some scholars have conducted research on tourism competitiveness in a provincial region, revealing that the essence of tourism destination competitiveness is the comprehensive quality of tourism development in different regions (Zhang and Zhou, 2005; Wen and Liang, 2007; Zhang et al., 2011).

The formation and development of tourism competitiveness are affected by many factors. For example, Eraqi (2011) assessed the influencing factors of tourism competitiveness from different aspects, and discussed in depth the importance of Egyptian enterprises’ marketing strategies and their tourism management attitudes toward tourism competitiveness. Finally, he analyzed ways to better implement these aspects in the Egyptian tourism industry to make full use of the potential of tourism competitiveness, finding the suitability of a marketing strategy for this purpose (Law and Ting, 2011). Mayaka and Akama (2007), in their study in Kenya, reported that the key factor for tourism competitiveness is the quality of human resources in Kenya’s tourist destinations. Mihalič (2000) reported that tourist destinations, tourist routes, and the tourism industry are the main factors to use when measuring tourism competitiveness. Scholars have found that the main influencing factors of tourism competitiveness are human resources, talent competition, image marketing, and knowledge-based management (Wei, 2000; Nie, 2006; Zhang et al., 2011; Cvelbar et al., 2016; Yang et al., 2020; Lan et al., 2021). In addition, tourists’ perceptions and the level of scientific and technological development of tourist destinations have a significant impact on the competitiveness of urban tourism destinations.

Mountain tourism is considered to be a vital factor for economic development and forms the soul of the tourism industry (Chin et al., 2014). This adds to the competition within the mountain tourism industry. Assessment of the competitiveness of mountain tourism has a pivotal role in the sustainable development of this industry (Lo et al., 2019). Appropriate assessment can help to develop this industry into becoming a competitive environment. Although mountain tourism may not be a top contributor in the mainstream economy, it makes a valuable contribution to the mountain economy in a large country like China. Previous studies have focused on mountain tourism as a way to generate income, support local communities, and create local employment (Wang et al., 2013; Shi et al., 2016; Mendola and Volo, 2017; Shen et al., 2019). However, research is lacking on the assessment of mountain tourism competitiveness. Therefore, this study intends to fill the research gap by developing a model for assessment of tourism competitiveness and testing the model in a case in the Western mountains of China. This study adopts all available indicators from prior studies and customizes them in the EAP context.

## Materials and Methods

### Study Area

The Enshi Autonomous Prefecture (EAP) of China is full of natural resources; its climate, topography, vegetation, and other features are distinctive, as are its tourism opportunities. The expansion of the tourism sector is a critical component of the southwestern region’s economic prosperity. As a result, the current study’s research focus is southwestern China (EAP).

**FIGURE 1:**
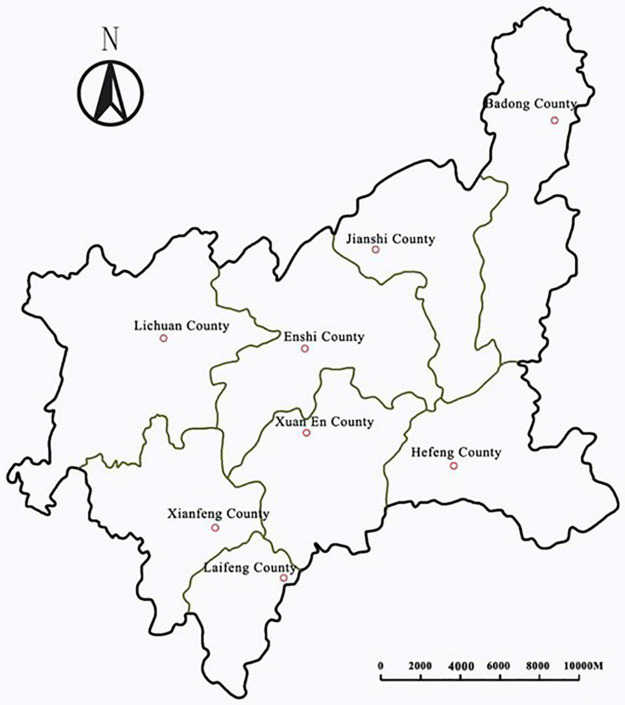
Regional distribution map of eight counties in Enshi Autonomous Prefecture (EAP), China.

Enshi Autonomous Prefecture is located in the southwest of Hubei Province. It is a typical mountainous area; thus, it is mainly mountain tourism. The prefecture’s territory covers an area of 24,000 km^2^. It comprises eight counties: Enshi County, Lichuan County, Jianshi County, Badong County, Xuanen County, Laifeng County, Xianfeng County, and Hefeng County, as shown in Figure 1. The registered population is 4,020,000, among whom the population of minorities accounts for 54%. In the 1990s, EAP’s mountain tourism industry began to develop gradually. By the end of 2016, 31 A-level scenic spots were designated: mentionable among them were two scenic spots at 5A level and 16 scenic spots above 4A level. A-level scenic spots rank in the forefront of Hubei Province, with EAP also having 46 hotels at higher than three-star level. Among travel agencies, 13 are above 3A level, of which two are at 4A level. Accommodation is provided by, among others, 75 star-rated hotels. Tour guides holding tour guide qualification certificates number 1,558. Mountain tourism leads, directly, to the employment of more than 100,000 people and, indirectly, leads to the employment of 400,000 people (Cao et al., 2019).

### Methods

Factor analysis was used in this study; specifically, the study used a multivariate statistical method that originated from Karl Pearson and Charles Spearman’s statistical analysis of psychological tests in the early 20th century. This method’s core is to use the least independent factors to reflect the vast majority of the information of the original variables. Through the analysis of the causal relationships between indicators, researchers are able to find out the main contradictions and key indicators.

#### Factor Analysis Model

Let, *P* observable indicators be *X*_1_, *X*_2_, *X*_3_, …, *X*_*p*_. The unobservable factors are *F*_1_, *F*_2_, *F*_3_, …, *F*_*m*_. The factor analysis model is described as follows:

$$X_{1}=a_{11}\,F_{1}+a_{12}\,F_{2}+\ldots +a_{1\,m}\,F_{m}+\varepsilon_{1}$$

$$X_{2}=a_{21}\,F_{1}+a_{22}\,F_{2}+\ldots +a_{2\,m}\,F_{m}+\varepsilon_{2}$$

…⁢…

Xp=ap1⁢F1+ap2⁢F2+…+ap⁢m⁢Fm+εp

m<p

The common factor of *X* is called *F*. Its mean vector *E*(*F*) = 0 and for the covariance matrix Cov (*F*) = 1. Therefore, each component of the vector ε(*ε_1_*,ε_2_,…, *ε_*p*_*) is independent of each other, which is a special factor. It is independent of *F*, and *E*(*e*) = 0.

*A* = (*a*_*ij*_), *a*_*ij*_ is the factor load, with it possible to be proved mathematically that the factor load *a*_*ij*_ is the correlation coefficient between index *I* and factor *J*. If the load is larger, this influences the closeness of the relationship between the *j* index and the *I* factor; conversely, the smaller the load, the more distant the relationship.

#### Factor Analysis Steps

##### Standardization of Raw Data

The non-dimensionalization of indicators is used to transform different indicators into uniform relative values through mathematical transformation, eliminating the influence of the different dimensions of each indicator.

$$Z_{i}=\frac{x_{i}-{\bar{x}}_{i}}{\sqrt{\frac{1}{N}\,\sum^{n}{\left(x_{i}-{\bar{x}}_{i}\right)}^{2}}},\left(i=1,2,\ldots ,N\right)$$

##### Computation of Eigenvalues

In accordance with the eigenvalue equation |*R*−*E*| = 0, the eigenvalues λ and corresponding eigenvectors A, λ of the correlation matrix are calculated. The sizes of the eigenvalues describe the role of each factor in interpreting the object.

##### Factor Contribution Rate

The factor contribution rate represents the ratio of the degree of variation of each factor to the degree of variation of all factors. The formula is as follows:

$$C_{i}=\lambda_{i}/\sum_{i=1}^{P}\lambda_{i}$$

*C*_*i*_ denotes the contribution rate of variance. When the cumulative contribution rate is over 85% or the characteristic root λ is not less than 1, the number of common factors is determined.

##### Factor Load Matrix

With *X* = AF, the factor load matrix A is not unique, but this study uses different parameter estimation methods to obtain the corresponding estimation matrix. The parameter estimation methods mainly include the following: least squares, maximum likelihood, principal component, principal factor, and multiple regression.

If the factor load is relatively average, the initial factor load matrix meaning will not be properly shown. It is difficult to judge the relationship between factors if rotation in the factors is required. The contribution of common factors after rotation is more dispersed by factor rotation, which mainly includes the two methods of orthogonal rotation and oblique rotation.

The factor score coefficient matrix B of the factors is obtained through the factor load matrix. The score *F* = BZ of each factor is then calculated. Finally, the weight of the variance contribution rate of each factor to the total variance of the factor is taken as the weighted sum, and the comprehensive score is obtained.

$$F=\frac{\lambda_{1}}{\sum_{i=1}^{m}\lambda_{i}}\,F_{1}+\frac{\lambda_{2}}{\sum_{i=1}^{m}\lambda_{i}}\,F_{2}+\ldots +\frac{\lambda_{m}}{\sum_{i=1}^{m}\lambda_{i}}\,F_{m}$$

By using the factor module calculation function of the analysis software, IBM SPSS Statistics (v. 20.0), the entire process of factor analysis can be quickly completed. Therefore, this study evaluates EAP’s tourism competitiveness through the use of SPSS software.

### Data Variables

The success of an objective and scientific evaluation of regional tourism competitiveness depends on the rationality and integrity of the index system design. In this study, the selection of evaluation indicators for EAP’s mountain tourism competitiveness mainly referred to the existing literature, both from China and internationally, on the evaluation of tourism competitiveness (Leong and Tan, 1992; Kozak and Rimmington, 1999; Kim et al., 2000; Wei, 2000; Zhou et al., 2002; Zhang and Zhou, 2005; Nie, 2006; Sánchez et al., 2006; Zhang and Lu, 2012), and was carried out using systematic analysis and screening.

In strict accordance with the basic principles of rationality and effectiveness, this study combines the characteristics of EAP’s mountain tourism industry development. A multi-dimensional index system is established, which consists of three first-level indicators and 18 second-level indicators (presented in Table 1). The structure of the evaluation index system of EAP’s mountain tourism competitiveness is as follows:

**TABLE 1:** Evaluation index system of EAP mountain tourism competitiveness.

Destination layer	Criteria layer	Index layer
EAP’s mountain tourism competitiveness (A)	Core competitiveness of tourism (A_1_)	Comprehensive tourism income (B1) Tourist reception numbers (B2) Domestic tourist reception numbers (B3) Number of travel agencies (B4) Number of star-rated hotels (B5) Number of star-rated rooms (B6) A-score of tourism resources (B7)
	Economic environment’s competitiveness (A_2_)	Gross domestic product (GDP) (B8) Third industry output value (B9) Fixed assets investment of entire society (B10) Financial expenditure (B11) Financial revenue (B12) Per capita disposable income of urban residents (B13) Per capita disposable income of mountain residents (B14)
	Infrastructure competitiveness (A_3_)	Highway mileage (B15) Highway density (B16) Passenger and freight transport volume (B17) Passenger turnover (B18)

Core competitiveness of tourism:

$$\,{\text{A}}_{1}=\left({\text{B}}_{1},{\text{B}}_{2},{\text{B}}_{3},{\text{B}}_{4},{\text{B}}_{5},{\text{B}}_{6},{\text{B}}_{7}\right)$$

Economic environment0s competitiveness:

$$\,{\text{A}}_{2}=\left({\text{B}}_{8},{\text{B}}_{9},{\text{B}}_{10},{\text{B}}_{11},{\text{B}}_{12},{\text{B}}_{13},{\text{B}}_{14}\right)$$

Infrastructure competitiveness: A_3_ = (B_15_, B_16_, B_17_, B_18_)

### Data Sources

By consulting the *EAP Statistical Yearbook*, EAP’s mountain tourism data from 2005 to 2014 were collected. From 2005 to 2014, the mountain tourist reception numbers, comprehensive income from tourism, foreign exchange income, domestic and foreign tourist reception numbers, travel agencies, star-rated hotels, A-level scenic spots, star-rated hotel rooms, etc. showed a growing trend. Among these data, the mountain tourist reception numbers increased by 2,163,300–31,004,100, indicating an increase of 17.52%; the comprehensive income from mountain tourism increased from 159 to 201 million yuan, indicating an increase of 10.26%; the number of travel agencies increased by 42; while the number of star-rated scenic spots increased by 27. Following the structural requirements of the evaluation index system for EAP’s mountain tourism competitiveness, EAP’s tourism data from 2005 to 2014 were systematically combed and analyzed, as shown in Table 2.

**TABLE 2:** Indicators of EAP mountain tourism competitiveness.

Indicators	Enshi County	Lichuan County	Laifeng County	Badong County	Xianfeng County	Jianshi County	Hefeng County	Xuanen County
B_1_	761,313	385,693	49,041	313,800	304,336	105,108	43,253	37,582
B_2_	10,694,446	7,325,123	988,847	4,841,620	3,632,040	1,680,501	963,940	877,629
B_3_	10,679,253	7,323,661	988,847	4,503,269	36,320,40	1,680,390	963,940	877,629
B_4_	43	8	3	0	3	5	0	0
B_5_	31	17	1	14	4	4	2	2
B_6_	3,147	2,161	86	1,028	405	933	167	120
B_7_	30	25	6	24	8	8	4	6
B_8_	1,565,030	904,681	528,189	8,14,511	603,784	709,908	435,522	500,584
B_9_	679,461	353,565	257,149	307,495	269,990	293,219	157,604	216,255
B_10_	1,366,674	919,882	513,494	785,547	527,737	727,928	438,534	375,078
B_11_	289,606	386,868	195,769	299,573	144,889	269,872	186,726	201,808
B_12_	234,974	151,769	68,596	77,165	57,656	84,755	35,720	36,896
B_13_	22,142	20,092	19,398	19,123	18,919	19,018	19,231	18,870
B_14_	7,453	7,091	7,050	7,140	7,077	7,145	7,546	7,048
B_15_	2311.5	3865.51	1112.53	3455.89	1861.5	2275.65	2041.32	1855.01
B_16_	30.24	87.71	45.5	81.56	61.29	54.87	101.46	61.55
B_17_	943	581	494	414	197	241	227	184
B_18_	58,153	35,820	30,458	25,519	12,156	14,889	14,022	11,371

*Source: EAP Statistical Yearbook 2005–2014.*

## Results

### Standardization of Data Processing

To make the data comparable and reflect the relative position of the indicators, standardization was used to eliminate the influence of the original indicators from the different kinds of raw data. Finally, the analysis was carried out as shown in Table 3.

**TABLE 3:** Standardized results of EAP mountain tourism competitiveness indicators.

Indicators	Enshi County	Lichuan County	Laifeng County	Badong County	Xianfeng County	Jianshi County	Hefeng County	Xuanen County
B_1_	1.89	0.68	−1.01	0.32	0.27	−0.73	−1.04	−1.07
B_2_	1.29	1.32	−1.1	0.37	−0.09	−0.84	−1.11	−1.14
B_3_	1.3	1.35	−1.1	0.26	−0.08	−0.83	−1.11	−1.14
B_4_	2.59	0.02	−0.35	−0.57	−0.35	−0.2	−0.57	−0.57
B_5_	1.75	0.95	−1.05	0.58	−0.67	−0.67	−0.92	−0.92
B_6_	1.25	1.46	−1.16	0.03	−0.76	−0.09	−1.06	−1.12
B_7_	0.61	1.35	−0.96	1.23	−0.71	−0.71	−1.2	−0.96
B_8_	2.22	0.49	−0.77	0.19	−0.52	−0.16	−1.08	−0.86
B_9_	2.38	0.27	−0.44	−0.07	−0.34	−0.17	−1.17	−0.74
B_10_	1.78	0.85	−0.77	0.31	−0.71	0.08	−1.07	−1.32
B_11_	−1.22	1.75	−0.64	0.66	−1.28	0.29	−0.75	−0.56
B_12_	1.7	1.19	−0.51	−0.33	−0.73	−0.18	−1.18	−1.16
B_13_	2.33	0.56	−0.23	−0.54	−0.77	−0.66	−0.42	−0.83
B_14_	1.79	−0.51	−0.71	−0.27	−0.58	−0.24	1.74	−0.72
B_15_	−1.55	1.51	−1.23	1.11	−0.48	−0.07	−0.31	−0.49
B_16_	−2.12	0.82	−0.74	0.59	−0.16	−0.39	1.32	−0.15
B_17_	1.79	0.85	0.42	0.02	−1.06	−0.84	−0.91	−1.12
B_18_	1.8	0.85	0.41	0.02	−1.06	−0.84	−0.91	−1.12

### Analysis of the Core Competitiveness of Tourism

Seven of the indicators that affect the core competitiveness of the EAP tourism industry underwent the Kaiser–Meyer–Olkin (KMO) test and Bartlett’s test of sphericity. Judging of the correlation coefficients and partial correlation coefficients was used to determine if these indicators were suitable for factor analysis. If the KMO statistic was greater than 0.5 and less than 1, these seven indicators could be used for factor analysis. The closer the value of the KMO statistic was to 1, the more suitable it would be as a factor.

With the KMO value of 0.547 at 5% level of significance, this indicates that the core competitiveness index data are suitable for factor analysis (Table 4).

**TABLE 4:** Kaiser–Meyer–Olkin (KMO) test and Bartlett’s test of sphericity.

Kaiser–Meyer–Olkin (KMO) measure of sampling sufficiency		0.547
Bartlett’s test of sphericity	Approximate chi-square value	90.762

	*df*	21
	Sig.	0.000

*df, degrees of freedom; Sig., significance.*

The study uses factor analysis to analyze the correlation coefficient matrix of EAP’s core competitiveness and the eigenvalues, variance contribution rate, and cumulative contribution rate are calculated. From the seven indicators, this study chooses an eigenvalue greater than 1 with a common factor to ensure the method’s validity and reliability. This reveals that the eigenvalue is 6.030, and the cumulative variance contribution rate is 86.139%. Shi et al. (2016) obtained almost similar results (79.7%) for tourism competitiveness in the border counties of Liaoning and Guangxi Province of China. This common factor properly reflects the level of EAP’s tourism core competitiveness (Table 5).

**TABLE 5:** Total variance of interpretation.

Component	Initial eigenvalue			Extract squared sum loading		
	Total	Variance %	Accumulated %	Total	Variance %	Accumulated %
1	6.030	86.139	86.139	6.030	86.139	86.139
2	0.699	9.992	96.131			
3	0.152	2.174	98.305			
4	0.101	1.436	99.741			
5	0.012	0.166	99.907			
6	0.006	0.093	100.000			
7	1.073E-5	0.000	100.000			

As shown in Table 5, each index value’s load for the common factor is obtained, and the size of the common factor is then calculated according to the load of each index. The public factors are mainly determined by the following seven indicators: comprehensive tourism income; number of mountain tourist receptions; number of domestic tourist receptions; number of travel agencies; number of star-rated hotels; number of star-rated rooms; and A-score of tourist attractions. The revenue can be raised for specific tourism if more tourists visit there (Liu et al., 2021). When tourism revenue grows faster, local tourism income rises, residents’ and staff’s income rises, local economic development improves, and the competitiveness of mountain-based other factors also rises (Zeng et al., 2021). The load of these seven indicators on the public factors is 0.958, 0.984, 0.983, 0.739, 0.984, 0.948, and 0.875, respectively, as expressed in F_1_.

The formula for calculating the common factor F_1_ is obtained from the component coefficient matrix, as shown in Table 6.

$$F_{1}=\,0.159\,B_{1}+0.163\times B_{2}+0.163\times B_{3}+0.123\times B_{4}+0.163\times B_{5}+0.157\times B_{6}+0.145\times B_{7}$$

**TABLE 6:** Component score coefficient matrix.

Component	Value
B_1_	0.159
B_2_	0.163
B_3_	0.163
B_4_	0.123
B_5_	0.163
B_6_	0.157
B_7_	0.145

Among them, B_1_, B_2_, B_3_, B_4_, B_5_, B_6_, and B_7_ are the variables of the original data after standardization. The mountain tourism core competitiveness of EAP is expressed by the public factor F_1_, from which the score of A_1_ can be obtained for EAP’s mountain tourism core competitiveness and the ranking of its core competitiveness, as shown in Table 7.

**TABLE 7:** Scores and rankings of EAP’s tourism core competitiveness (A_1_).

Region	A_1_	Ranking
Enshi County	1.61	1
Lichuan County	1.13	2
Jianshi County	−0.64	5
Badong County	0.36	3
Xuanen County	−1.08	7
Xianfeng County	−0.36	4
Laifeng County	−1.05	6
Hefeng County	−1.09	8

The score reflects the size of EAP’s mountain tourism core competitiveness. The values in Table 7 show that EAP ranks the core competitiveness of tourism in its counties from strong to weak, in descending order, from Enshi County, Lichuan County, Badong County, Xianfeng County, Jianshi County, Laifeng County, Xuanen County, and Hefeng County. Enshi County has the highest score among the eight counties, with its core tourism competitiveness value being 1.61.

### Analysis of Economic Environment’s Competitiveness

Using the factor analysis module of SPSS (v. 20.0), the standardized EAP economic environment’s competitiveness index is analyzed, with the factor analysis model’s validity judged using the KMO test and Bartlett’s test of sphericity. The KMO value is 0.562 which is higher than 0.5 at the 10% significance level, but less at the 5% significance level. These indicators, shown in Table 8 below, meet the requirements for factor analysis (Table 8).

**TABLE 8:** Kaiser–Meyer–Olkin (KMO) test and Bartlett’s test of sphericity.

Kaiser–Meyer–Olkin (KMO) measure of sampling sufficiency		0.562
Bartlett’s test of sphericity	Approximate chi-square value	63.573

	*df*	21
	Sig.	0.000

*df, degrees of freedom; Sig., significance.*

The factor analysis of EAP’s economic environment’s competitiveness is carried out, and the eigenvalues, variance contribution rate, and cumulative contribution rate are calculated as shown in Table 9. According to the principle that the eigenvalue is greater than 1, two common factors, namely, F_2_ and F_3_, are selected for the seven indicators of EAP’s economic environment’s competitiveness. The variance contribution rates of the two common factors are 68.921 and 21.722%, respectively. The cumulative variance contribution rate is 90.642%, and the eigenvalues are 4.887 and 1.458, respectively. These two public factors reflect the competitiveness of EAP’s economic environment (Table 9).

**TABLE 9:** Total variance of interpretation.

Component	Initial eigenvalue			Extract squared sum loading			Rotating squared sum loading		
	Total	Variance %	Accumulated%	Total	Variance %	Accumulated%	Total	Variance %	Accumulated%
1	4.887	69.818	69.818	4.887	69.818	69.818	4.824	68.921	68.921
2	1.458	20.824	90.642	1.458	20.824	90.642	1.521	21.722	90.642
3	0.507	7.243	97.885						
4	0.119	1.695	99.580						
5	0.024	0.337	99.917						
6	0.004	0.063	99.980						
7	0.001	0.020	100.000						

The competitiveness of EAP’s economic environment has two public factors, namely F_2_ and F_3_. On the one hand, F_2_ is mainly determined by gross domestic product (GDP). The tertiary industry’s output value, investment in fixed assets, fiscal revenue, urban residents’ per capita disposable income, and mountain residents’ per capita disposable income. Their principal component loads are 0.983, 0.967, 0.958, 0.952, 0.953, and 0.498, respectively. On the other hand, F_3_ is mainly focused on fiscal expenditure, with the load on the principal component being 0.918 (Table 10).

**TABLE 10:** Component score coefficient matrix.

	Component	
Factor	1	2
B_8_	0.159	0.036
B_9_	0.163	−0.066
B_10_	0.163	0.232
B_11_	0.123	0.918
B_12_	0.163	0.255
B_13_	0.157	−0.179
B_14_	0.145	−0.677

As shown in Table 10, the scoring matrix of the two common factor components is calculated as follows:

$$F_{2}=\,0.203\times B_{8}+0.190\times B_{9}+0.216\times B_{10}+0.217\times B_{11}+0.101\times B_{12}+0.176\times B_{13}+0.038\times B_{14}$$

$$F_{3}=\,0.003\times B_{8}+0.072\times B_{9}-0.131\times B_{10}-0.147\times B_{11}-0.622\times B_{12}+0.148\times B_{13}+0.474\times B_{14}$$

The scores and rankings of the two public factors of EAP’s economic environment’s competitiveness, namely, F_2_ and F_3_, are then used to determine A_2_, using the formula below, as listed in Table 11.

$$A_{2}=\left(68.921/90.642\%\right)\times F_{2}+\left(21.722/90.642\%\right)\times F_{3}$$

**TABLE 11:** Scores and rankings of EAP’s economic environment’s competitiveness (A_2_).

Region	F_2_	Ranking	F_3_	Ranking	A_2_	Ranking
Enshi County	1.67	1	0.26	4	1.34	1
Lichuan County	0.91	2	−1.25	8	0.40	2
Jianshi County	−0.13	4	−0.17	7	−0.14	4
Badong County	0.10	3	−0.14	6	0.04	3
Xuanen County	−1.01	8	0.46	2	−0.66	8
Xianfeng County	−0.83	6	0.32	3	−0.56	7
Laifeng County	−0.66	5	0.11	5	−0.48	6
Hefeng County	−0.96	7	1.66	1	−0.33	5

According to the comprehensive score, namely, A_2_, of the two public factors, this study reveals that EAP’s economic and environmental competitiveness rankings are as follows, in descending order: Enshi County > Lichuan County > Badong County > Jianshi County > Hefeng County > Laifeng County > Xianfeng County > Xuanen County. Of these, Enshi County has the strongest economic and environmental competitiveness, scoring 1.34, while Xuanen County has the weakest economic and environmental competitiveness, scoring −0.66. Zeng et al. (2022) argue that the economic environment is a key dimension of mountain tourism development. The competitive market fails to distribute resources efficiently to overcome the unfavorable consequences. As a result, worldwide cooperation is required to keep mountain tourism alive as a tourist savior (Nguyen et al., 2022).

### Analysis of Infrastructure Competitiveness

Through the KMO test and Bartlett’s test of sphericity, this study determines that the value of the KMO statistic is 0.524 which is greater than 0.5 at the 10% significance level, but less at the 5% significance level. Therefore, the EAP’s infrastructure competitiveness index can be analyzed by factor analysis (Table 12).

**TABLE 12:** KMO test and Bartlett’s test of sphericity.

Kaiser–Meyer–Olkin (KMO) measure of sampling sufficiency		0.524
Bartlett’s test of sphericity	Approximate chi-square value	58.506

	*df*	6
	Sig.	0.000

*df, degrees of freedom; Sig., significance.*

The study uses SPSS to analyze the factor values in the correlation coefficient matrix of EAP’s infrastructure competitiveness. The eigenvalues, variance contribution rates, and cumulative contribution rates are also calculated (Table 13). To ensure the validity and reliability of the method, this study chooses an eigenvalue greater than 1, with two common factors, namely, F_4_ and F_5_, focusing on four indicators of EAP’s infrastructure competitiveness. The variance contribution rates of these two common factors are 53.523 and 42.365%, respectively. The cumulative variance contribution rate of 95.887% is greater than an eigenvalue of 1. Therefore, these two factors can be used as public factors to reflect the competitiveness of EAP’s infrastructure.

**TABLE 13:** Total variance of interpretation.

Component	Initial eigenvalue			Extract squared sum loading			Rotating squared sum loading		
	Total	Variance %	Accumulated%	Total	Variance %	Accumulated%	Total	Variance %	Accumulated%
1	2.530	63.258	63.258	2.530	63.258	63.258	2.141	53.523	53.523
2	1.305	32.630	95.887	1.305	32.630	95.887	1.695	42.365	95.887
3	0.165	4.113	100.000						
4	1.019E-5	0.000	100.000						

As shown in Table 14, the scoring matrix of the two common factor components is calculated as follows:

$$F_{4}=0.190\times B_{15}-0.071\times B_{16}+0.486\times B_{17}+0.486\times B_{18}$$

$$F_{5}=0.635\times B_{15}+0.472\times B_{16}+0.089\times B_{17}+0.88\times B_{18}$$

$$A_{3}=\left(53.523/95.887\%\right)\times F_{4}+\left(42.365/95.887\%\right)\times F_{5}$$

**TABLE 14:** Component score coefficient matrix.

	Component	
Factor	1	2
B_15_	0.190	0.635
B_16_	−0.071	0.479
B_17_	0.486	0.089
B_18_	0.486	0.088

This study obtains the scores and rankings of the public factors of EAP’s infrastructure competitiveness, in particular, the size of EAP’s infrastructure competitiveness A_3_ (Table 15).

**TABLE 15:** Scores and rankings of EAP’s infrastructure competitiveness (A_3_).

Region	F_4_	Ranking	F_5_	Ranking	A_3_	Ranking
Enshi County	1.60	1	−1.68	8	0.15	3
Lichuan County	1.05	2	1.5	1	1.25	1
Jianshi County	−0.80	5	−0.38	4	−0.61	6
Badong County	0.19	4	0.99	2	0.54	2
Xuanen County	−1.17	8	−0.58	6	−0.91	8
Xianfeng County	−1.11	7	−0.57	5	−0.87	7
Laifeng County	0.22	3	−1.06	7	−0.35	4
Hefeng County	−1.03	6	0.28	3	−0.45	5

As shown in Table 15, the descending order of EAP’s infrastructure competitiveness is Lichuan County > Badong County > Enshi County > Laifeng County > Hefeng County > Jianshi County > Xianfeng County > Xuanen County. The counties with strong infrastructure competitiveness are Lichuan, Badong, and Enshi, measured as 1.25, 0.54, and 0.15, respectively. From a competition point of view, the competitiveness of Lichuan County, Badong County, and Enshi County is higher due to the long mileage and high road density of these three counties. Lo et al. (2019) measured the destination competitiveness of rural tourism in Malaysia and reported that the infrastructure of the tourist place has a vital effect on attracting tourist and competitiveness of the industry. Yang et al. (2020) argue that to prolong tourist travel, enhance tourism consumption, and raise tourism revenue, it is required to strengthen tourism infrastructure construction. Similarly, Reisinger et al. (2019) argue that infrastructure is important but not sufficient for tourism, and its success is dependent on several other factors.

### Analysis of Enshi Autonomous Prefecture’s Mountain Tourism Competitiveness

The proportion of variances to total variances is determined, using the weighted method, from the results of EAP’s mountain tourism core competitiveness, economic and environmental competitiveness, and infrastructure competitiveness. These weighted values are 84.292, 13.4, and 2.308%, respectively (Table 16).

**TABLE 16:** Total variance of interpretation.

Component	Initial eigenvalue			Extract squared sum loading		
	Total	Variance %	Accumulated %	Total	Variance %	Accumulated %
1	2.529	84.292	84.292	2.529	84.292	84.292
2	0.402	13.400	97.692			
3	0.069	2.308	100.000			

This study also obtained EAP’s mountain tourism competitiveness A by weighting the scores of EAP’s mountain tourism core competitiveness, economic and environmental competitiveness, and infrastructure competitiveness (Table 17).

$$A=0.84292\times A_{1}+0.134\times A_{2}+0.02308\times A_{3}$$

**TABLE 17:** Scores and rankings of EAP’s mountain tourism competitiveness.

Region	A_1_	A_2_	A_3_	A	Ranking
Enshi County	1.61	1.34	0.15	1.54	1
Lichuan County	1.13	0.4	1.25	1.03	2
Badong County	0.36	0.04	0.54	0.32	3
Xianfeng County	−0.36	−0.56	−0.87	−0.40	4
Jianshi County	−0.64	−0.14	−0.61	−0.57	5
Laifeng County	−1.05	−0.48	−0.35	−0.96	6
Hefeng County	−1.09	−0.33	−0.45	−0.97	7
Xuanen County	−1.08	−0.66	−0.91	−1.02	8

The values in Table 17 show that the mountain tourism competitiveness of each county in EAP is quite different. Enshi County, Lichuan County, and Badong County have positive scores in tourism competitiveness, indicating that their tourism competitiveness is relatively strong and has competitive advantages. However, five counties, namely, Xianfeng County, Jianshi County, Laifeng County, Hefeng County, and Xuanen County, had negative scores in tourism competitiveness, indicating that these five counties had weak tourism competitiveness. Enshi County’s score of 1.54 indicates that it has the strongest tourism competitiveness while Xuanen County’s score of −1.02 indicates that it has the weakest competitiveness. Although each county is in the same region, the development of the tourism industry across the counties is not balanced. The descending order of tourism competitiveness is Enshi County > Lichuan County > Badong County > Xianfeng County > Jianshi County > Laifeng County > Hefeng County > Xuanen County. Enshi County, Lichuan County, and Badong County are strong in tourism core competitiveness, economic and environmental competitiveness, and infrastructure competitiveness, while Jianshi County, Xianfeng County, Xuanen County, Laifeng County, and Hefeng County have weak competitiveness.

Therefore, EAP’s mountain tourism competitiveness is quite different in terms of the respective contributions of tourism core competitiveness, economic and environmental competitiveness, and infrastructure competitiveness. The findings are almost similar to other studies that assessed mountain tourism (Bacoş and Gabor, 2021; Gao et al., 2021; Zeng et al., 2021; Zeng et al., 2022). The main contribution to EAP’s mountain tourism competitiveness is tourism core competitiveness, followed by economic and environmental competitiveness, and, finally, infrastructure competitiveness. When tourism core competitiveness is increased by one unit, EAP’s mountain tourism competitiveness will increase by 0.84292 units. Similarly, when economic environment competitiveness is increased by one unit, EAP’s mountain tourism competitiveness will increase by 0.134 units. When infrastructure competitiveness increases by one unit, EAP’s mountain tourism competitiveness increases by 0.02308 units. Michael et al. (2019) claim that tourism competitiveness is influenced by destination resources, destination infrastructure and support services, and the general business environment in United Arab Emirates. Similarly, Cvelbar et al. (2016) argue that tourism infrastructure and destination management are the primary competitiveness drivers in developing countries, whereas destination competitiveness in developed countries is influenced by tourism-specific factors like infrastructure, economic, and business environment.

## Discussion

### A Large Gap Exists in Tourism Competitiveness Between Enshi Autonomous Prefecture’s Counties

Enshi County has the highest score for mountain tourism competitiveness (1.54), while Xuanen County has the lowest (−1.02); thus, the competitiveness gap between Enshi County and Xuanen County is 2.56. Analysis of these model values shows that the level of Enshi County’s tourism competitiveness gap is large, with obvious polarization and excessively strong cooperation. Therefore, this weakens the basis for cooperation between counties, leading to ineffective overall cooperation in EAP’s mountain tourism industry. There are numerous benefits of mountain tourism, including the development of suburban rural tourism, many issues eventually arise that limit its growth. Mountain tourism may be an essential driver for rural development in remote mountain villages that confront major economic, social, and environmental issues, according to case studies from Germany, Italy, Romania, Ukraine, and Poland (Lun et al., 2016). As a result, retail trade, transportation, and communication strategies should focus on maintaining natural and cultural resources more successfully. A sufficient local, employable population is critical in establishing mountain tourism locations with greater employment prospects (Zeng et al., 2022).

### The Degree of Tourism Cooperation Between Enshi Autonomous Prefecture’s Counties Is Low

The degree of tourism cooperation focuses on the extent of relationships between two or more actors who agree to share information, technical assistance, management training, capital, and/or market intelligence, either formally or informally (Reisinger et al., 2019). Interorganizational connections impact the entire local system in tourism (Li and Du, 2021). An autonomous organization interacts to generate joint actions, utilizing common rules, conventions, and structures to deliberate and act on issues linked to the region’s tourism growth (Dias et al., 2021). Public and private organizations are active in interorganizational collaboration to promote the tourism destination’s long-term viability and competitiveness. The inter-organizational linkages extend to tourist management agencies, which help managers, company owners, and government officials develop communication channels and narrow interpersonal interactions (Gao et al., 2021). Publicity for Enshi County’s Mountain tourism product is self-centered. Even though it formulates mountain tourism routes as a unit, the mountain tourism products of various regions have not been effectively combined. Most existing high-quality tourist routes are designed around scenic spots in Enshi County, Lichuan County, Badong County, and Jianshi County, with the tourist attractions of Xianfeng County, Xuanen County, Hefeng County, and Laifeng County not listed. Even though some scenic spots in Jianshi County and Badong County are listed in the high-quality tourist routes, insufficient cooperation is evident. Therefore, tourism competition between EAP’s counties is greater than tourism cooperation, with the degree of cooperation between counties rated as low. Wilke et al. (2019) argue that organizational learning is facilitated by accessing new sources of information, which aids in the development of dynamic capacities in the tourism industry.

### Lack of a Sense of Cooperation Between Enshi Autonomous Prefecture’s Counties

Cooperation between organizations is critical for enterprises to gain valuable resources like information and knowledge, commodities and services, finance, markets, and technology (Luštický and Štumpf, 2021). Cooperation has been emphasized in particular in the context of tourist destinations, where ties and integration between companies involved in tourism on a direct or indirect basis contribute to providing consumers with a comprehensive tourist experience (Nguyen et al., 2022). In essence, research indicates that the tourism business would not reach its full potential without cooperation amongst many stakeholders (Wu et al., 2022). Currently, no sense of cooperation is found between EAP’s counties. Cooperation in EAP’s mountain tourism industry is still at a low level, with the importance of cooperation not really understood in terms of ideology and action. Each county in EAP is fighting for its own independent development and its own competitiveness, so it is difficult for them to reach consensus and form a joint force. Therefore, it is necessary to elevate the cooperative consciousness of EAP’s mountain tourism, form an industrial cluster of mountain tourism, and bring into play the cooperative effect of “1 + 1 > 2.” According to Wilke et al. (2019), organizations create a variety of talents through collaboration, which results in sustained competitive advantage and higher performance.

### Imperfect Infrastructure in Enshi Autonomous Prefecture’s Counties

The analysis of EAP’s infrastructure competitiveness shows a large gap. The tourism competitiveness of each county is closely related to its infrastructure construction. Due to the mountainous area, road construction is difficult and costly, with EAP having few tourist highways. Although the main highway has been built between counties, the quality of the highway is not at the same standard and the road is narrow, contributing to the failure of effective cooperation between counties. At present, only Enshi County, Lichuan County, Jianshi County, and Badong County have railways. Existing transportation facilities cannot meet the needs of tourists, which greatly hinders EAP’s ability to attract foreign tourists. Bazargani and Kiliç (2021) argue that the infrastructure is a fundamental driver of tourism success, but policy conditions, the institutional framework, and socio-cultural resources are all important drivers. Similarly, Lim et al. (2019) report that infrastructures alone are insufficient yet crucial aspects that attract visitors to tourism destinations and meet their needs once they arrive at a competitive destination.

## Conclusion

Benign interaction between competition and cooperation is the driving force for the healthy development of EAP’s mountain tourism industry. To effectively overcome the problems from tourism competition between EAP’s counties, solutions need to achieve “win-win” cooperation within the competition. It is found that Enshi county is more competitive in terms of tourism than other seven counties under EAP. This study explores that core competitiveness, economic and environmental competitiveness, and infrastructural competitiveness are worth 84.29, 13.4, and 2.31%, respectively. EAP’s mountain tourism competitiveness rises by 0.84 units when tourism core competitiveness rises by one unit. Similarly, when the competitiveness of the economic environment improves by one unit, the competitiveness of EAP’s mountain tourism improves by 0.134 units. EAP’s mountain tourist competitiveness grows by 0.023 units when infrastructural competitiveness increases by one unit. Lack of awareness of the county administration, a low level of cooperation, and poor infrastructure were the main causes of low competitiveness.

The recommendations from this study’s findings are as follows. Firstly, it is necessary to properly handle the relationship between competition and cooperation, maintaining cooperation in competition and competition in cooperation, and promoting the healthy development of EAP’s regional mountain tourism industry. Secondly, county authorities should strengthen communication, establish an effective coordination mechanism, achieve effective regional coordination and benefit distribution, and jointly promote the development of EAP’s mountain tourism industry. Thirdly, the sense of cooperation must be enhanced and jointly developed within the mountain tourism market. For example, EAP could formulate joint mountain tourism image promotional plans, with the slogan “EAP’s Wonderland” to develop the overall mountain tourist market. Fourthly, the construction of tourism infrastructure must be strengthened to break down the barriers to tourism cooperation. Strengthening the construction of transportation infrastructure is the first priority for EAP’s mountain tourism competition and cooperation. Traffic links between county towns and scenic spots are also necessary to strengthen, rebuild, and optimize existing roads, and ensure the smooth flow of traffic on mountain tourist roads in each county’s area. Road network links between different counties and scenic spots should be strengthened to promote the coordinated development of the mountain tourism industry involving all counties. This study’s findings will be helpful for developing a cooperation mechanism and sustainable development, thus reducing poverty and promoting the mountain revitalization of China. The results of this study are applicable for mountain regions worldwide in general and China in particular.

## Data Availability Statement

The original contributions presented in the study are included in the article/supplementary material, further inquiries can be directed to the corresponding author.

## Author Contributions

QC designed the research plan, collected and analyzed the data, and wrote the manuscript. MNIS, DZ, JS, and JD analyzed the data and revised the manuscript. TX collected and analyzed the data and revised the manuscript. All authors have checked the final version of the manuscript and approved it for publication.

## Conflict of Interest

The authors declare that the research was conducted in the absence of any commercial or financial relationships that could be construed as a potential conflict of interest.

## Publisher’s Note

All claims expressed in this article are solely those of the authors and do not necessarily represent those of their affiliated organizations, or those of the publisher, the editors and the reviewers. Any product that may be evaluated in this article, or claim that may be made by its manufacturer, is not guaranteed or endorsed by the publisher.

## References

[B1] Bacoş I. B. Gabor M. R. (2021). Tourism economy. Mountain tourism: quantitative analysis of winter destinations in romania. *Economics* 9 143–159. 10.2478/eoik-2021-0005

[B2] Bazargani R. H. Z. Kiliç H. (2021). Tourism competitiveness and tourism sector performance: empirical insights from new data. *J. Hosp. Tour. Manag.* 46 73–82. 10.1016/j.jhtm.2020.11.011

[B3] Cao Q. Sarker M. N. I. Sun J. (2019). Model of the influencing factors of the withdrawal from rural homesteads in China: application of grounded theory method. *Land Use Policy* 85 285–289. 10.1016/j.landusepol.2019.04.013

[B4] Chen C. M. Chen S. H. Lee H. T. (2011). The destination competitiveness of Kinmen’s tourism industry: exploring the interrelationships between tourist perceptions, service performance, customer satisfaction and sustainable tourism. *J. Sustain. Tour.* 19 247–264. 10.1080/09669582.2010.517315

[B5] Chin C.-H. Lo M.-C. Songan P. Nair V. (2014). Rural tourism destination competitiveness: a study on annah rais longhouse homestay, Sarawak. *Proc. Soc. Behav. Sci.* 144 35–44. 10.1016/j.sbspro.2014.07.271

[B6] Chon K. Li G. Lin S. Gao Z. (2010). Recovery of tourism demand in hong kong from the global financial and economic crisis. *J. China Tour. Res.* 6 259–278. 10.1080/19388160.2010.503916

[B7] Cvelbar L. K. Dwyer L. Koman M. Mihalič T. (2016). Drivers of destination competitiveness in tourism. *J. Travel Res.* 55 1041–1050. 10.1177/0047287515617299

[B8] Deng T. Hu Y. Ma M. (2019). Regional policy and tourism: a quasi-natural experiment. *Ann. Tour. Res.* 74 1–16. 10.1016/j.annals.2018.10.001

[B9] Dias Á González-Rodríguez M. R. Patuleia M. (2021). Retaining tourism lifestyle entrepreneurs for destination competitiveness. *Int. J. Tour. Res.* 23 701–712. 10.1002/jtr.2436

[B10] Enright M. J. Newton J. (2004). Tourism destination competitiveness: a quantitative approach. *Tour. Manag.* 25 777–788. 10.1016/j.tourman.2004.06.008

[B11] Eraqi M. I. (2011). Co-creation and the new marketing mix as an innovative approach for enhancing tourism industry competitiveness in Egypt. *Int. J. Serv. Oper. Manag.* 8:76. 10.1504/IJSOM.2011.037441 35009967

[B12] Gao J. Shao C. Chen S. Wei Z. (2021). Evaluation of sustainable development of tourism cities based on sdgs and tourism competitiveness index: analysis of 221 prefecture-level cities in China. *Sustainability* 13:12338. 10.3390/su132212338

[B13] Greene F. J. Tracey P. Cowling M. (2007). Recasting the city into city-regions: place promotion, competitiveness benchmarking and the quest for urban supremacy. *Growth Change* 38 1–22. 10.1111/j.1468-2257.2007.00350.x

[B14] Haahti A. J. (1986). Finland’S competitive position as a destination. *Ann. Tour. Res.* 13 11–35.

[B15] Jiao X. He R. Song W. Li J. (2018). Measurement and difference analysis on tourism competitiveness of Blue Economic Zone. *IOP Conf. Ser. Mater. Sci. Eng.* 423:012004. 10.1088/1757-899X/423/1/012004

[B16] Kim S.-S. Crompton J. L. Botha C. (2000). Responding to competition: a strategy for Sun/Lost City. *S. Afr. Tour. Manag.* 21 33–41. 10.1016/S0261-5177(99)00094-1

[B17] Kozak M. Rimmington M. (1999). Measuring tourist destination competitiveness: conceptual considerations and empirical findings. *Int. J. Hosp. Manag.* 18 273–283. 10.1016/S0278-4319(99)00034-1

[B18] Lan F. Huang Q. Zeng L. Guan X. Xing D. Cheng Z. (2021). Tourism experience and construction of personalized smart tourism program under tourist psychology. *Front. Psychol.* 12:691183. 10.3389/fpsyg.2021.691183 34367015 PMC8339922

[B19] Law R. (2001). Mix, match & move: shaping the future of tourism Asia pacific tourism association seventh annual conference Manila, the Philippines, 2001. *Asia Pac. J. Tour. Res.* 6 74–75. 10.1080/10941660108722101

[B20] Law R. (2007). A fuzzy multiple criteria decision-making model for evaluating travel websites. *Asia Pac. J. Tour. Res.* 12 147–159. 10.1080/10941660701243372

[B21] Law R. Ting J. (2011). The impact of visitor behavior on the environmental protection of tourist farms in Guangdong. *Asia Pac. J. Tour. Res.* 16 307–323. 10.1080/10941665.2011.573244

[B22] Lee C. F. Huang H. I. Yeh H. R. (2010). Developing an evaluation model for destination attractiveness: sustainable forest recreation tourism in Taiwan. *J. Sustain. Tour.* 18 811–828. 10.1080/09669581003690478

[B23] Leong S. M. Tan C. T. (1992). Assessing national competitive superiority: an importance-performance matrix approach. *Mark. Intell. Plan.* 10 42–48. 10.1108/02634509210007902

[B24] Li S. Du S. (2021). An empirical study on the coupling coordination relationship between cultural tourism industry competitiveness and tourism flow. *Sustainability* 13 1–14. 10.3390/su13105525

[B25] Lim C. Zhu L. Koo T. T. R. (2019). Urban redevelopment and tourism growth: relationship between tourism infrastructure and international visitor flows. *Int. J. Tour. Res.* 21 187–196. 10.1002/jtr.2253

[B26] Liu M. Li Y. Pérez-Sánchez M. Á Luo J. Bu N. Chen Y. (2022). Empirical study on the sustainable development of mountain tourism in the early stage of high-speed railways—taking the southwest mountainous region of china as an example. *Sustainability* 14:1058. 10.3390/su14031058

[B27] Liu Y. L. Ko P. F. Chiang J. T. (2021). Developing an evaluation model for monitoring country-based tourism competitiveness. *SAGE Open* 11 1–18. 10.1177/21582440211047559

[B28] Lo M.-C. Chin C.-H. Law F.-Y. (2019). Tourists’ perspectives on hard and soft services toward rural tourism destination competitiveness: community support as a moderator. *Tour. Hosp. Res.* 19 139–157. 10.1177/1467358417715677

[B29] Lopes A. P. F. Muñoz M. M. Alarcón-Urbistondo P. (2018). Regional tourism competitiveness using the PROMETHEE approach. *Ann. Tour. Res.* 73 1–13. 10.1016/j.annals.2018.07.003

[B30] Lun L.-M. Pechlaner H. Volgger M. (2016). Rural tourism development in mountain regions: identifying success factors, challenges and potentials. *J. Qual. Assur. Hosp. Tour.* 17 389–411. 10.1080/1528008X.2015.1096754

[B31] Luštický M. Štumpf P. (2021). Leverage points of tourism destination competitiveness dynamics. *Tour. Manag. Perspect.* 38:100792. 10.1016/j.tmp.2021.100792

[B32] Maso L. D. Liberatore G. Fazzini M. (2016). “Tourism destination competitiveness and firm performance through a financial crisis,” in *Tourism Management, Marketing, and Development*, eds Mariani M. M. Czakon W. Buhalis D. Vitouladiti O. (New York, NY: Palgrave Macmillan US).

[B33] Mayaka M. Akama J. S. (2007). Systems approach to tourism training and education: the Kenyan case study. *Tour. Manag.* 28 298–306. 10.1016/j.tourman.2005.12.023

[B34] Mendola D. Volo S. (2017). Building composite indicators in tourism studies: measurements and applications in tourism destination competitiveness. *Tour. Manag.* 59 541–553. 10.1016/j.tourman.2016.08.011

[B35] Michael N. Reisinger Y. Hayes J. P. (2019). The UAE’s tourism competitiveness: a business perspective. *Tour. Manag. Perspect.* 30 53–64. 10.1016/j.tmp.2019.02.002

[B36] Mihalič T. (2000). Environmental management of a tourist destination. *Tour. Manag.* 21 65–78. 10.1016/S0261-5177(99)00096-5

[B37] Nazmfar H. Eshghei A. Alavi S. Pourmoradian S. (2019). Analysis of travel and tourism competitiveness index in middle-east countries. *Asia Pac. J. Tour. Res.* 24 501–513. 10.1080/10941665.2019.1590428

[B38] Nguyen T. Q. T. Johnson P. Young T. (2022). Networking, coopetition and sustainability of tourism destinations. *J. Hosp. Tour. Manag.* [Epub ahead of print] 10.1016/j.jhtm.2022.01.003

[B39] Nie X. (2006). Structural analysis and competitiveness study of urban tourism attraction. *Mod. Urban Res.* 1 81–83. 10.3923/itj.2013.2905.2912

[B40] Patton S. G. (1985). Tourism and local economic development: factory outlets and the reading SMSA. *Growth Change* 16 64–73. 10.1111/j.1468-2257.1985.tb00758.x

[B41] Pearce D. G. (1997). Competitive destination analysis in Southeast Asia. *J. Travel Res.* 35 16–24. 10.1177/004728759703500403

[B42] Peng C. Yuan P. (2019). Influence of environmental regulations on China’s tourism competitiveness. *Nankai Bus. Rev. Int.* 10 429–446. 10.1108/NBRI-12-2017-0073

[B43] Reisinger Y. Michael N. Hayes J. P. (2019). Destination competitiveness from a tourist perspective: a case of the United Arab Emirates. *Int. J. Tour. Res.* 21 259–279. 10.1002/jtr.2259

[B44] Ritchie J. R. B. Crouch G. I. (2000). The competitive destination: a sustainability perspective. *Tour. Manag.* 21 1–7. 10.1215/00703370-8937033 33834242

[B45] Sánchez J. Callarisa L. Rodríguez R. M. Moliner M. A. (2006). Perceived value of the purchase of a tourism product. *Tour. Manag.* 27 394–409. 10.1016/j.tourman.2004.11.007

[B46] Sedlacek S. Zekan B. Weismayer C. Gunter U. Schuh B. (2022). Regional sustainability and tourism carrying capacities. *J. Clean. Prod.* 339:130624. 10.1016/j.jclepro.2022.130624

[B47] Shen S. Wang H. Quan Q. Xu J. (2019). Rurality and rural tourism development in China. *Tour. Manag. Perspect.* 30 98–106. 10.1016/j.tmp.2019.02.006

[B48] Shi Y. Zhong L. Chen T. Yu H. (2016). Tourism competitiveness evaluation and spatio-temporal characteristics of Chinese border counties. *Chinese Geogr. Sci.* 26 817–828. 10.1007/s11769-016-0822-1

[B49] Su W. Yang Y. Gu C. (2003). A preliminary study on the evaluation of urban tourism competitiveness. *J. Tour.* 3 39–42.

[B50] Wang L. Cheng S. Zhong L. Mu S. Dhruba B. G. C. Ren G. (2013). Rural tourism development in China: principles, models and the future. *J. Mt. Sci.* 10 116–129. 10.1007/s11629-013-2501-3

[B51] Wei X. (2000). Development goals and knowledge competition of China’s tourism. *Soc. Sci.* 1 4–14.

[B52] Wen B. Liang M. (2007). Study on the evaluation model of regional tourism competitiveness based on factor analysis. *J. Tour.* 22 18–22.

[B53] Wilke E. P. Costa B. K. Freire O. B. D. L. Ferreira M. P. (2019). Interorganizational cooperation in tourist destination: building performance in the hotel industry. *Tour. Manag.* 72 340–351. 10.1016/j.tourman.2018.12.015

[B54] Wu M. Y. Wu X. Li Q. C. Tong Y. (2022). Community citizenship behavior in rural tourism destinations: scale development and validation. *Tour. Manag.* 89:104457. 10.1016/j.tourman.2021.104457

[B55] Yang J. Wang J. Zhang L. Xiao X. (2020). How to promote ethnic village residents’ behavior participating in tourism poverty alleviation: a tourism empowerment perspective. *Front. Psychol.* 11:2064. 10.3389/fpsyg.2020.02064 33013524 PMC7461915

[B56] Zeng L. Li R. Y. M. Huang X. (2021). Sustainable mountain-based health and wellness tourist destinations: the interrelationships between tourists’ satisfaction, behavioral intentions, and competitiveness. *Sustainability* 13:13314. 10.3390/su132313314

[B57] Zeng L. Li R. Y. M. Nuttapong J. Sun J. Mao Y. (2022). Economic development and mountain tourism research from 2010 to 2020: bibliometric analysis and science mapping approach. *Sustainability* 14:562. 10.3390/su14010562

[B58] Zhang F. Sarker M. N. I. Lv Y. (2022). Coupling coordination of the regional economy, tourism industry, and the ecological environment: evidence from western China. *Sustainability* 14:1654. 10.3390/su14031654

[B59] Zhang H. Gu C. L. Gu L. W. Zhang Y. (2011). The evaluation of tourism destination competitiveness by TOPSIS & information entropy - A case in the Yangtze River Delta of China. *Tour. Manag.* 32 443–451. 10.1016/j.tourman.2010.02.007

[B60] Zhang Y. Lu L. (2012). “On the Tourism competitiveness of scenic spots based on fuzzy comprehensive evaluation method,” in *Information Engineering and Applications Lecture Notes in Electrical Engineering*, eds Zhu R. Ma Y. (London: Springer London), 1223–1228.

[B61] Zhang Z. Zhou Y. (2005). An empirical study on urban tourism competitiveness: guangdong province as an example. *Resour. Dev. Mark.* 21 13–16.

[B62] Zhou S. Chen S. Shao J. (2002). A comparative study of urban tourism competitiveness in Xi’an. *J. Northwest Univ.* 32 103–106.

